# Atypical Presentation and Diagnosis of Multiple Myeloma: A Case Report

**DOI:** 10.7759/cureus.51870

**Published:** 2024-01-08

**Authors:** Iris Kong, Luke W Riddell, Jacquelyn M Kohler, Michael C Nguyen, Michelle N Carraro

**Affiliations:** 1 Department of Emergency and Hospital Medicine, Lehigh Valley Health Network/University of South Florida (USF) Morsani College of Medicine, Allentown, USA

**Keywords:** imaging, blunt trauma, multiple myeloma, expansile mass, vertebral artery dissection

## Abstract

Chronic neck pain is a common, seemingly benign condition that typically does not warrant an urgent workup, in contrast to acute onset neck pain. Vertebral artery dissection (VAD) is a relatively rare presentation of acute onset neck pain and often presents in the context of blunt trauma. Due to the risk of subsequent clot formation and stroke, patients who present with symptoms suggestive of VAD must be promptly screened, most often first with computed tomography angiography (CTA) followed by magnetic resonance imaging (MRI) or magnetic resonance angiography (MRA) for further evaluation. We present a case of a 69-year-old male with a history of chronic neck pain who was seen in the emergency department due to acute, severe neck pain with initial CTA imaging that suggested left VAD. However, follow-up MRI of his cervical spine identified that what seemed to be a left VAD was instead multiple myeloma. This case demonstrates the utility of using multiple imaging modalities, including CT, CTA, MRI, and MRA, in diagnosing an atypical presentation of multiple myeloma and the consequences of physician implicit biases that are often involved when treating patients with chronic pain.

## Introduction

Neck pain is a common condition that may not only cause substantial distress and disability for the individual but also significantly impact healthcare systems and economic costs globally [1]. One cause of severe neck pain that must always be considered in an acute setting, due to its gravity, is vertebral artery dissection (VAD). VAD arises from compromised arterial wall integrity and blood invasion into the tunica media through an intimal tear in the vertebral artery [2]. Aggregation of blood in the arterial wall promotes hematoma and clot formation, leading to stenosis, VAD symptoms, and stroke. VADs are rare causes of stroke, with an incidence of about 1-5 per 100,000 and more commonly present a few days after an acute event, most often blunt trauma [2]. Other precipitating factors may include neck manipulation, such as in chiropractic procedures, and sudden movements, such as coughing or vomiting [2]. Risk factors predisposing individuals to VADs include connective tissue disorders such as Ehlers-Danlos syndrome, female gender, fibromuscular disorders, oral contraceptive usage, vasculitis, and the postpartum period [3]. Patients with VAD may present with symptoms including headache, nausea, ataxia, dysarthria, lateral medullary syndrome, coma, subarachnoid hemorrhage, spinal cord infarction, and cervical nerve root impairment [2,3].

On radiographic imaging, VADs are most often located in the V2 or V3 segments of the vertebral artery and may involve the intradural portion of V4 and the origin of the posterior inferior cerebellar artery [2]. Computed tomography angiography (CTA) may show irregularity of the arterial lumen, such as a characteristic “double lumen” or thickening of the arterial wall [2]. Magnetic resonance imaging (MRI) and magnetic resonance angiography (MRA) offer higher sensitivity and specificity for identifying ischemia and intramural hemorrhage, as well as for imaging vessel lumens; these imaging modalities are often used after CTAs to better elucidate the etiology and evaluate the VADs [2].

Multiple myeloma accounts for 1.6% of all cancer cases in the United States [4]. Patients may present with nonspecific symptoms such as nausea, vomiting, weakness, bone pain, or malaise, and it is not uncommon for patients to be asymptomatic, with the initial diagnosis being made via laboratory abnormalities [4]. Multiple myeloma can most frequently be identified in imaging studies by characteristic bone or marrow lesions [5]. Invasion of soft tissues on initial presentation is rare due to multiple myeloma’s origin in the marrow.

We present a case in which a patient's chronic neck pain clouded the clinical picture of his new acute onset neck pain, which ultimately required multiple imaging modalities to elucidate the patient's final diagnosis of multiple myeloma, initially presumed to be an acute VAD. This case report, in part, was previously presented as an abstract and displayed in an image gallery at the 2023 Pennsylvania College of Emergency Physicians Scientific Assembly (May 5, 2023, Pocono Manor, Pennsylvania).

## Case presentation

A 69-year-old male with a history of 35 years of tobacco use, hyperlipidemia, fibromyalgia, chronic back pain, benign prostatic hypertrophy, gastroesophageal reflux disease, diverticulitis, kidney stones, and type II diabetes mellitus presented to the emergency department (ED) with sudden, severe burning neck pain. He had been experiencing constant neck pain for the past two months and visited the ED at another local institution four days prior due to similar symptoms of acute neck pain. There, he was diagnosed with spasmodic torticollis and discharged with recommendations to use prescription lidocaine transdermal patches, cyclobenzaprine hydrochloride, and follow-up with Orthopedics.

The next day, X-rays of his cervical spine displayed degenerative disc disease at C5 and C6 with multilevel facet joint disease. He was advised to obtain an outpatient MRI and continue with pain control and antispasmodics. However, he was awoken again by severe neck pain that morning. The patient denied recent fevers, chest pain, lower back pain, nausea, vomiting, diarrhea, numbness, seizures, syncope, tingling in his arms and legs, tremors, difficulty finding words, weakness, dysphagia, or dysarthria. Emergency medical services had administered ketorolac and fentanyl for his pain.

Upon physical examination, the patient was hypertensive but afebrile with normal respirations. He did not appear to be in acute distress or toxic. Pain with movement of the cervical spine and surrounding muscular tenderness were present, but he did not display spinous process tenderness, rigidity, or crepitus. He demonstrated a normal range of motion of his cervical back and 5/5 strength in all extremities.

Preliminary laboratory values were drawn, and initial complete blood count (CBC) with differential, comprehensive metabolic panel (CMP) and troponin I were sent. His CBC was within normal limits, except for a mildly elevated white count of 11.2 thou/cmm (4.5-11), mildly elevated absolute neutrophils of 9.7 thou/cmm (1.8-7.8), and decreased absolute lymphocyte count of 0.7 thou/cmm (1.0-3.0). His CMP demonstrated a normal anion gap, elevated glucose of 207 mg/dL (65-99), decreased sodium at 132 mmol/L (135-145), elevated potassium of 5.6 mmol/L (3.5-5.2), elevated BUN at 36 mg/dL (7-28), decreased albumin of 3.1 g/dL (3.5-4.8), elevated total protein of 10.7 g/dL (6.3-8.3), and elevated aspartate aminotransferase (AST) of 72 U/L (<41), with other components of the CMP remaining normal. His troponin I was within normal limits.

**Figure 1:**
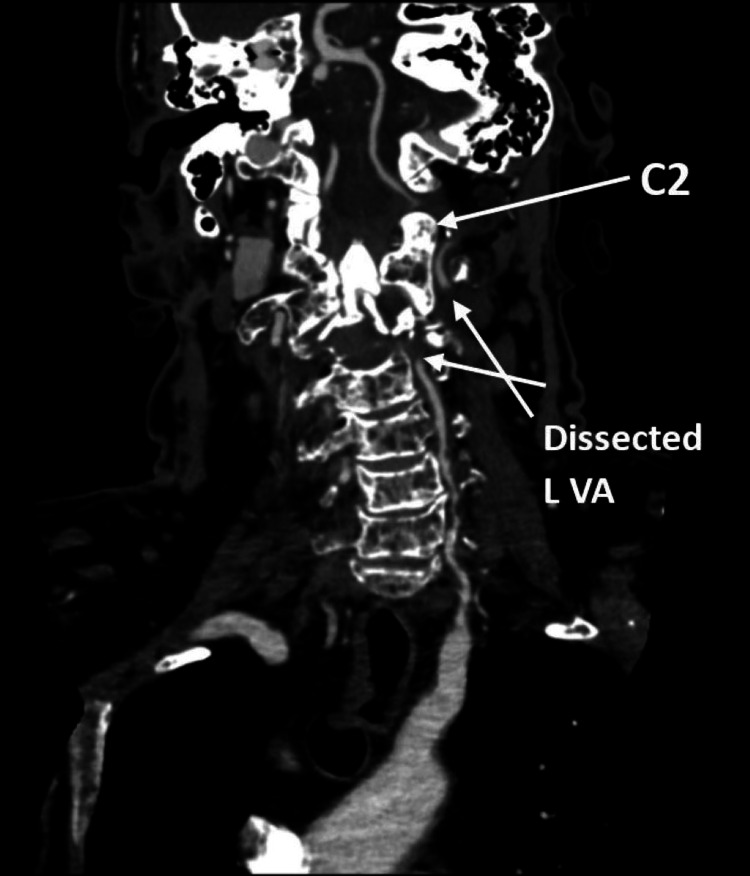
CT angiogram displaying an expansile lytic lesion of the C2 vertebral body and moderate, focal compression of the distal right V2 segment with severe stenosis of the proximal left V2.

**Figure 2:**
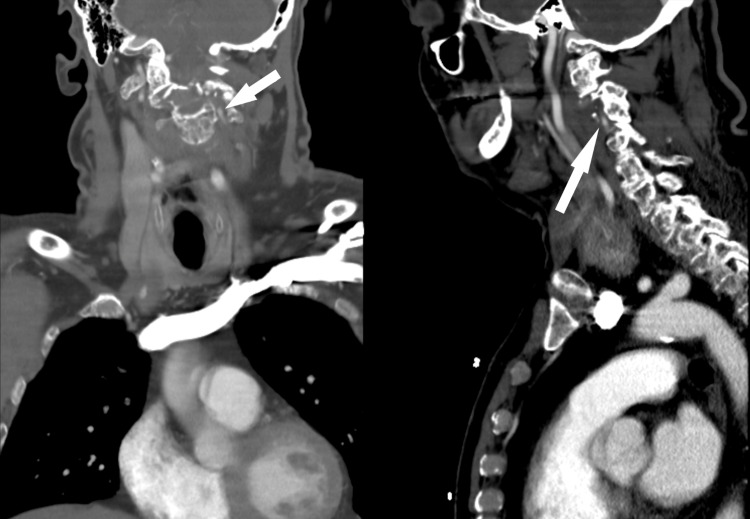
CT angiograms indicating the point of vertebral artery dissection and complete loss of contrast enhancement of the left vertebral artery at the C2 level. There is a long segment of narrowing with mural thickening of the left V3 segment indicative of a VA dissection, vessel comes to a tapering point as it is compressed by the pathological fracture of C2, and moderate focal extrinsic compression of the distal right V2 segment due to pathologic fracture of C2.

Initial imaging included computed tomography (CT) of the cervical spine, computed tomography angiography (CTA), magnetic resonance angiography (MRA) of the head and neck, and MRI of the brain and cervical spine. CT of the cervical spine and CTA illustrated an expansile lytic lesion of the C2 vertebral body with associated pathologic compression deformity. The CT and CTA also demonstrated moderate focal extrinsic compression of the distal right V2 segment due to a pathologic fracture of C2 and severe stenosis of the proximal left V2 with moderate narrowing of the mid-left V2. The complete loss of enhancement of the left vertebral artery at the C2 level with short segment reconstitution at the C1 level and long segment narrowing and mural thickening of the left V3 segment highly suggested dissection of the left vertebral artery (Figure 1). The coronal and sagittal CTA indicates the point of VA dissection (Figure 2).

**Figure 3:**
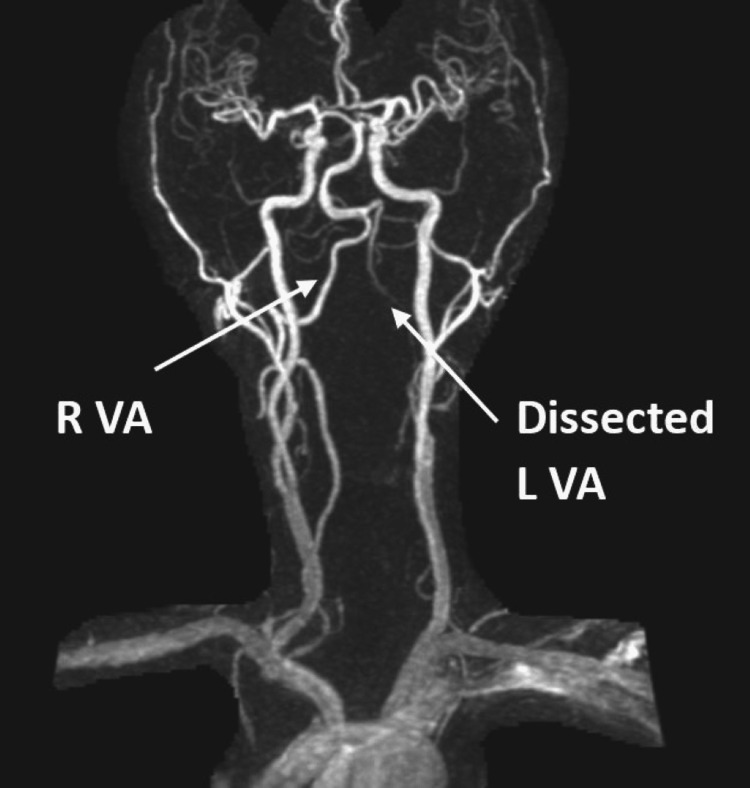
MRA of the head and neck illustrating no flow signal of the entirety of the left vertebral artery.

The MRA of the brain and neck demonstrated a lack of flow signal at the left vertebral artery with small, irregular left V1 and proximal to mid V2, and peripheral T1 hyperintense signaling at the left V2 (Figure 3). These MRA findings further supported the theory of left cervical VAD and possibly an intramural hematoma at V2.

**Figure 4:**
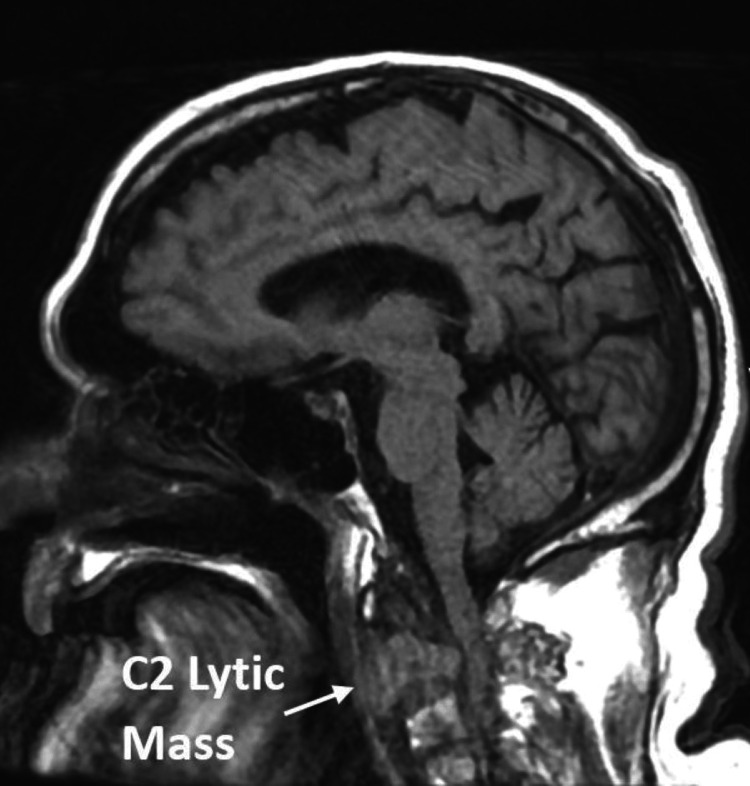
MRI of the cervical spine exhibiting the expansile marrow-displacing, enhancing process involving the C2 and odontoid process.

MRI of the brain and MRA of the brain and neck did not display any acute infarction or intracranial hemorrhage. However, MRI of the cervical spine illustrated an expansile marrow replacing enhancing process involving the C2 and odontoid that would cause severe spinal canal stenosis and compression of the spinal cord (Figure 4).

The results of the MRI of the cervical spine suggested a possible neoplastic/metastatic process. Interventional Radiology was consulted for a CT-guided biopsy of the mass. The patient’s CT chest with contrast demonstrated unremarkable lungs, pleura, heart, vessels, mediastinum, and lymph nodes. However, the chest wall and bones displayed mottled-appearing osseous structures with tiny lucencies diffusely scattered throughout the bony thorax and thoracic spine, possibly representing metastatic disease. For further workup, a core biopsy was obtained from C2, demonstrating a Kappa light chain restricted plasma cell neoplasm. Microscopic examination of the sections showed a diffuse population of plasma cells with a subset displaying atypical features, including larger size, more open chromatin, and variably present nucleoli. Immunohistochemical stains displayed atypical plasma cells positive for CD138, BCL1 (subset), and kappa, but negative for lambda. Interphase FISH was normal for all loci studied. Cytogenetics were performed months after treatment, all demonstrating a normal 46, XY male karyotype. After initiating dexamethasone with radiation therapy and wearing a neck brace, the patient reported less pain. The patient started daratumumab, bortezomib, lenalidomide, and dexamethasone (Dara-VRd) therapy following dexamethasone and radiation therapy and continued to report improvement in pain and mobility.

## Discussion

For this patient, the initial imaging studies and acute presentation strongly supported the preliminary diagnosis of VAD. VADs most commonly occur at levels C1 and C2 and typically present with acute onset headache and neck pain-symptoms that all seemed to align with our patient’s presentation. Although the patient denied any recent inciting injuries or trauma to the head and neck, common in up to 80% of cervical artery dissections, he did endorse chronic neck pain for years from falling off a tree and a car crash that occurred years ago. Due to a high suspicion of VAD, CTA was appropriately ordered for screening, while MRI and MRA were used for further evaluation and a detailed work-up. The gold standard diagnostic imaging study for VADs is cerebral angiography, typically illustrating focal dilation and proximal or distal stenosis [2]. However, because cerebral angiography is more invasive and a higher-risk procedure, CTA is more commonly used for screening when VAD is suspected [6]. A systematic review of observational studies found that in cases of symptomatic VADs where conventional angiography was the reference standard, CTA had a sensitivity of 100%, whereas MRA and Doppler ultrasonography exhibited sensitivities of 77% and 71%, respectively; however, the specificity of any imaging technique for symptomatic VADs is largely unstudied [7].

In contrast to the epidemiology of VAD, multiple myeloma more commonly presents in older patients with more indolent, smoldering symptoms related to the infiltration of plasma cells into bone and other organs, or kidney damage from immunoglobulin deposition [8]. These symptoms are commonly summarized as “CRAB” symptoms and include calcium elevation, renal insufficiency, anemia, and bone disease. Two diagnostic criteria must be met for the diagnosis of multiple myeloma: the patient must present with more than 10% clonal bone marrow plasma cells or biopsy-proven bony or extramedullary plasmacytoma, in addition to one or more myeloma-defining events [9]. The gold standard diagnostic study for multiple myeloma is typically MRI or fluorodeoxyglucose (FDG)-positron emission tomography (PET)/CT [10].

In this case, MRI of the cervical spine deduced the root cause of this patient’s presentation-multiple myeloma. Our patient met the diagnostic criteria for multiple myeloma based on his biopsy results, bone disease, and MRI findings. The patient’s atypical presentation of multiple myeloma, suspiciously similar to classic VAD based on symptoms and initial screening, necessitated the use of more detailed imaging techniques. This prompted us to consider multiple myeloma as a differential diagnosis and to undertake further work-up. Without additional, proper imaging and investigation, the patient might have simply been treated with anticoagulation for VAD, and his definitive diagnosis and treatment for multiple myeloma would have been significantly delayed. Delayed diagnosis of multiple myeloma can affect subsequent treatment and patient eligibility for management such as hematopoietic stem cell transplantation [8]. As with other cancers, delays in diagnosis negatively impact the patient’s 5-year odds of survival.

This case serves as an important reminder for physicians to not only promptly recognize the clinical presentation and imaging findings of VAD to immediately address them but also to do their due diligence for patients with chronic pain. Physicians should broaden their differentials and complete a thorough history, physical exam, and work-up to prevent misdiagnoses that would delay diagnosis and treatment and prevent mortality from other conditions. Patients who experience chronic pain may not be treated with the same seriousness or urgency as those who experience acute symptoms, and their experiences may be discounted.

In this case, the patient’s history of chronic pain, acute presentation, and initial imaging led to a delayed diagnosis of multiple myeloma. Additionally, healthcare providers’ own implicit biases, differences in patient/provider expectations, and cultural and linguistic disparities further feed into the negative stigmatization of patients with chronic pain, limit access to pain treatments, impact diagnostic decision-making, and iatrogenically create a negative feedback loop [11]. To combat disjointed care, providers must work together with their patients, listen to their patients’ lived experiences, and explore their perspectives that may better reduce bias and inequities and increase empathy and health outcomes [12,13].

## Conclusions

This case illustrates the utility of CTA and MRI in evaluating a VAD that, with systematic work-up and detailed imaging studies, surprisingly concluded with a diagnosis of multiple myeloma for the patient. Although neck pain due to multiple myeloma is an uncommon presentation in the ED compared to VADs, physicians should consider multiple myeloma in their differential diagnosis when evaluating patients' clinical presentation with chronic pain, imaging studies, and laboratory work-up. A complete understanding of a patient’s history and the course of action throughout their care cycle, even from a different institution, is important for timely diagnosis. Ultimately, physicians should investigate thoroughly through interdisciplinary collaboration to provide optimal care to patients and minimize any biases that could result in delayed or missed diagnoses.
